# A rare case of myxoma mimicking pulmonary thromboembolism: a case report

**DOI:** 10.3389/fcvm.2024.1433381

**Published:** 2024-08-27

**Authors:** Shokoufeh Hajsadeghi, Saeid Hosseini, Hossein Saidi, Shayan Mirshafiee

**Affiliations:** ^1^ Research Center for Prevention of Cardiovascular Disease, Institute of Endocrinology and Metabolism, Iran University of Medical Sciences, Tehran, Iran; ^2^ Heart Valve Disease Research Center, Rajaie Cardiovascular Medical and Research Center, Iran University of Medical Sciences, Tehran, Iran; ^3^ Department of Emergency Medicine, Assistant Professor of Emergency Medicine, Iran University of Medical Sciences, Tehran, Iran; ^4^ Department of Cardiology, School of Medicine, Tehran University of Medical Sciences, Tehran, Iran

**Keywords:** case report, myxoma, pulmonary embolism, cardiac tumor, cardiac surgery

## Abstract

Studies have shown that primary cardiac tumors are exceptionally rare and among them myxomas represents approximately half. Although majority of these benign tumors are located in left atrium, they can also emerge in the right cardiac chambers and also on cardiac valves. In rare instances, they may extend into the great vessels. In this case, we present a teenage girl with a past medical history of successfully cured acute lymphoblastic leukemia, who presented with progressive dyspnea and lower limbs edema. Based on history, laboratory, and echocardiographic evidences, she was initially treated for high-risk thromboembolism but after inefficient response, she underwent surgery, with a large right ventricular outflow tract myxoma being diagnosed.

## Introduction

Primary cardiac tumors are exceptionally rare, with studies reporting maximum incidence of 0.1% in unselected autopsies, with cardiac myxomas comprising approximately half of these cases (1, 2). While most of these benign tumors are located in the left atrium, they can also develop in right cardiac chambers and on cardiac valves, with rare instances of occurrence in multiple chambers like biatrial myxomas or even great vessels (3, 4).

These tumors can emerge at any age, although diagnoses are commonly made between the third and sixth decades of life. The clinical manifestation of these masses can vary based on their size and location (5). The primary treatment of myxomas is surgical removal; however, recurrence following resection can be more aggressive (6).

In a minority of cases where patients do not fulfill the Carney complex criteria, a history of non-endocrine malignancy may be present. Notably, these individuals appear to have a higher incidence of right heart myxomas, which can be mistaken for more prevalent conditions such as cardiac metastases (7–9).

In this case, we introduce a teenage girl with a history of cured acute lymphoblastic leukemia (ALL) who presented with worsening dyspnea and lower limbs edema. According to history, laboratory data, and echocardiographic findings, she was initially managed for high-risk pulmonary thromboembolism but ultimately a different diagnosis was determined.

## Case description

A 15-year-old female, with a history of ALL cured eight years ago, was presented at emergency unit of Rasoul Akram Hospital in Tehran following complaints of worsening dyspnea, lower extremities edema, and abdominal distension. She had a history of recent flu-like symptoms. Initial physical examination revealed a blood pressure of 116/69, heart rate of 129 (beats/minute), and oxygen saturation of 94%, prompting a cardiology consultation due to her malignancy history and clinical symptoms.

Initial laboratory findings also revealed white blood cell (WBC)= 10.57 (×1,000/mm^2^), hemoglobin (Hb)= 10.2 (g/dl), platelet = 440 (×1,000/mm^2^), and positive qualitative amounts of D-dimer and C-reactive protein (CRP) with quantitative results of 1,100 ng/ml and 19 mg/dl (with a cut-off of 8), respectively.

The four-chamber echocardiographic view revealed severe right ventricular (RV) enlargement, severe regurgitation of the tricuspid valve, akinesia of RV free wall with apical sparing (McConnell sign) (Supplementary Video-S1), and parasternal short axis view showed a large mass-like echo density in RVOT protruding into the main pulmonary artery (PA) (Supplementary Video-S2).

**Figure 1:**
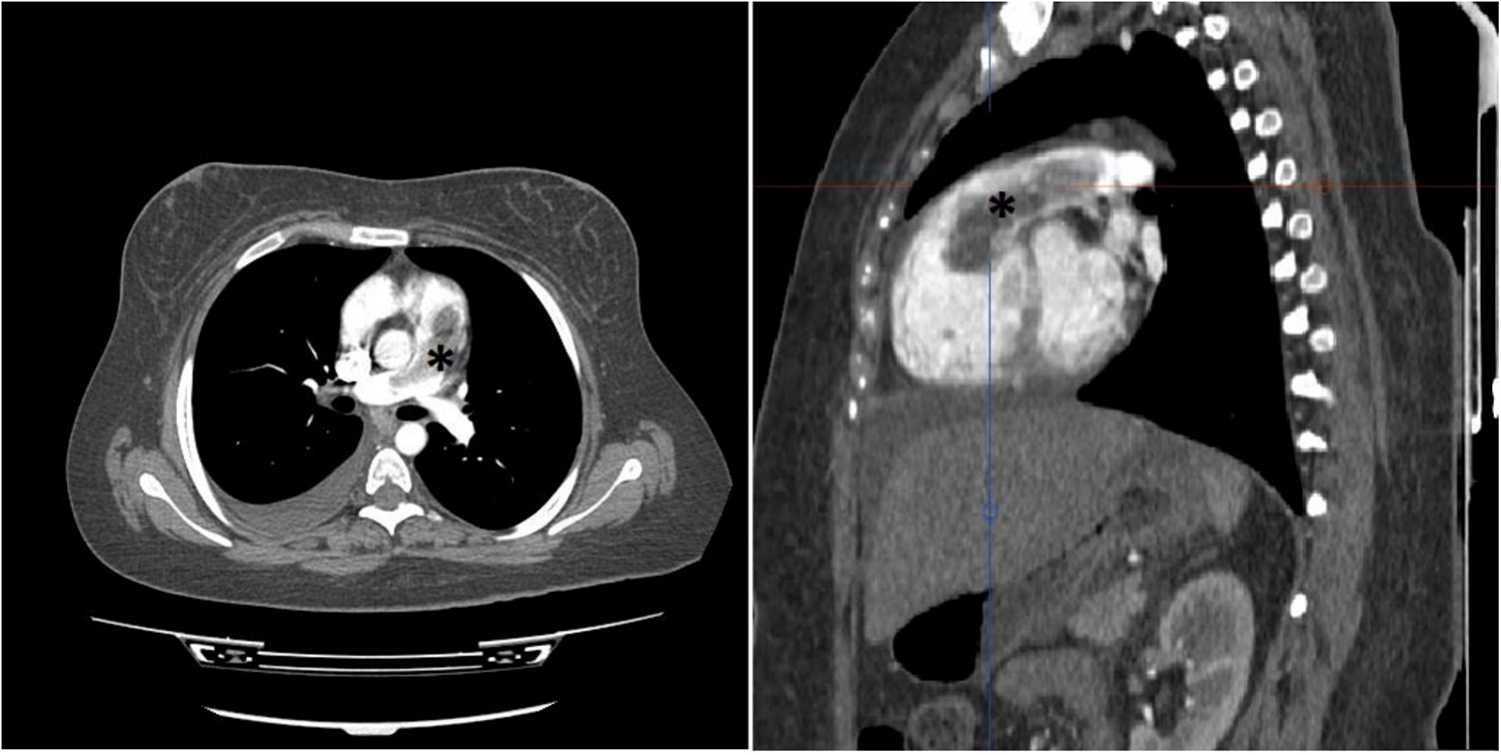
Thorax CT study with intravenous contrast in axial (left) and oblique sagittal (right) views shows elongated intraluminal mass and filling defect (*) arising from RVOT with extension to PA trunk. No invasion to myocardium is detected. No intraluminal filling defect in lobar and segmental branches of PA noted. (not shown).

In consideration of the patient's stable clinical status, an immediate computed tomography pulmonary angiography (CTPA) was performed, demonstrating severe RV dilatation with a large defect in the RVOT protruded to PA, while no defects were observed in more distal arterial branches (Figure 1).

Taking into consideration the clinical symptoms, borderline vital signs, history of cured malignancy, positive initial laboratory tests, echocardiographic findings, and the unfeasibility of emergency cardiac surgery upon the patient's arrival at the emergency unit, and also considering the absence of absolute or relative contraindications, thrombolytic was administered, followed by intensive care unit (ICU) monitoring. Subsequent echocardiography post-thrombolytic infusion indicated no improvement in RV function, leading to the decision for open surgery due to the persistent echo dense mass in the RVOT.

In the morning of scheduled surgery, the patient experienced hypotension and decreased O2 saturation, both of which were responsive to intravenous fluid and supplemental oxygen.

**Figure 2:**
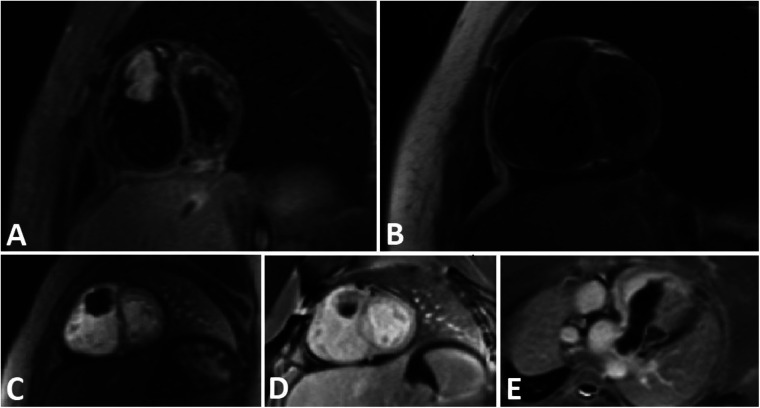
A tubular shape tumor in RVOT and protrusion in to the main PA. **(A)** Short axis view STIR sequence demonstrates high signal tumor. B) Short axis view T1-W sequence reveals Iso signal tumor. **(C)** Short axis view perfusion sequence shows no early enhancement. **(D,E)** Short axis and oblique view Late Gadolinium Enhancement (LGE) sequence demonstrates faint enhancement at some part of the tumor.

Cardiac magnetic resonance (CMR) imaging was conducted to investigate non-thrombotic causes (Figure 2), and subsequently, she was transferred to the operation room for surgical intervention.

**Figure 3:**
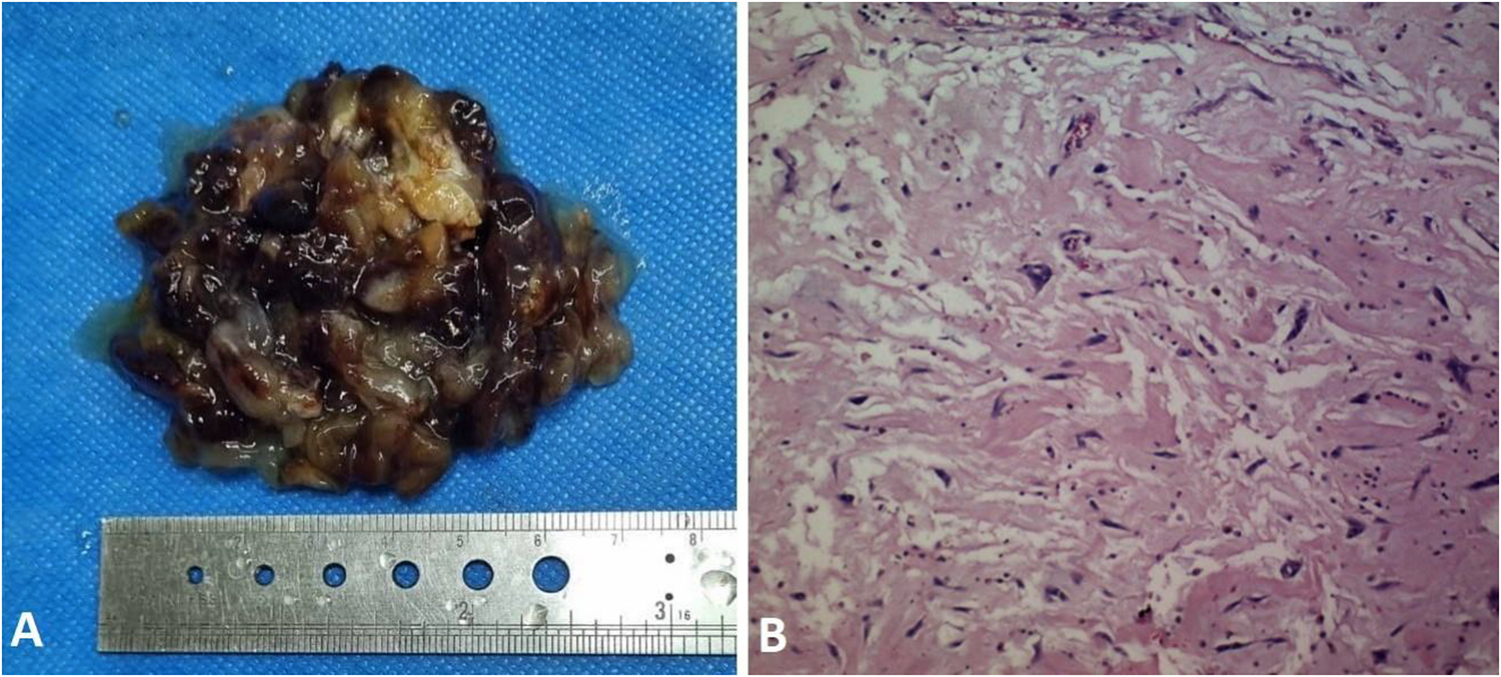
**(A)** sgross view of resected myxoma with maximum diameter of 7 centimeters. **(B)** Microscopic view of myxoma with polygonal and fusiform cells in myxoid matrix.

During the procedure, a large gelatinous mass, attached to the RVOT by a stalk, was identified and successfully resected (Figure 3). Following the surgery, the patient was transferred to ICU, maintaining stable clinical condition.

Upon histopathological examination confirming myxoma, postoperative echocardiography demonstrated enhanced RV function. Subsequently, she was discharged from the hospital in good general condition after one week.

## Discussion

Primary cardiac tumors, although rare and mostly benign, can provoke symptoms in patients due to their size and location. The most common variant, myxoma, primarily located in the left atrium (LA), may imitate symptoms of mitral valve stenosis, notably in the left lateral decubitus position. After the LA, the right atrium (RA) emerges as the second most common site for myxomas, representing approximately 15%–20% of cases. A minority of myxomas may also be found in the left or right ventricles. The identification of multiple myxomas or their presence in atypical locations should prompt consideration of familial disorders (5, 10–12).

In the presented case, the myxoma was situated in an exceedingly rare location—the RVOT with protrusion to main PA, leading to the initial misdiagnosis of high-risk thromboembolism for the patient.

Based on the literature review, the documented cases of myxoma in the RVOT and or pulmonary artery trunk are very few (13). Among the few reported cases, patients ages ranged from 11 to 58 years, with all cases exhibiting a maximum size of 2 centimeters, and no documented history of malignancy, often diagnosed incidentally (14–16). In our patient, the combination of a young age, malignancy history, symptoms of right heart failure, and a substantial 7 centimeters mass distinguished it from previous cases.

In the case report by Chun-Yao Huang et al., a 31-year-old man presented with respiratory distress, tachypnea, and tachycardia. Following a diagnosis of pulmonary embolism via echocardiography and CTPA, he was administered thrombolytics and discharged in stable condition. Surprisingly, he reappeared two weeks later with a relapse of symptoms. A large filling defect was detected in the CTPA, leading to the patient undergoing pulmonary endarterectomy. Subsequent confirmation of a pulmonary artery myxoma was made. Unfortunately, he passed away the next day due to severe RV dysfunction. They suggested that the thrombotic component was probably dissolved after thrombolytic infusion, but after two weeks and probable mass growth, the symptoms relapsed (17).

Although rapid mass growth within two weeks and normal coagulation parameters in their case do not support this argument, it is imperative to consider the possibility of simultaneous thrombosis and myxoma, with D-dimer negativity potentially providing diagnostic assistance (18). The higher-than-normal D-dimer level in our case did not provide clarity in ruling out pulmonary embolism.

Despite the lack of a family history of cardiac tumors or clinical features indicative of Carney complex in our case, the presence of a prior malignancy prompted a thorough investigation of comparable cases.

In the study by Alizadeh et al., conducted on breast cancer patients attending the cardio-oncology clinic, a higher prevalence of cardiac myxoma than expected was noted. This underscores the importance of follow-up echocardiographic monitoring and consideration of alternative diagnosis beyond thrombosis and metastasis (7).

In a separate instance, an elderly male with a history of treated colorectal cancer underwent echocardiography due to symptoms resembling those of our patient. A mobile non-homogenous mass attached to the anterior leaflet of the tricuspid valve was detected, leading to surgical intervention and the subsequent confirmation of myxoma (8).

The key similarity between our case and the two mentioned articles is the coexistence of myxoma in an atypical location and history of malignancy.

While a comprehensive study in this area is challenging due to the rarity of primary cardiac tumors, considering the diagnosis of myxoma in similar cases can be useful in choosing the best treatment approach.

## Conclusion

Although cardiac myxomas are rare in locations other than LA or RA, they should always be kept in mind, especially in patients with a history of malignancy. However, further investigations are required to confirm this assumption.

## Data Availability

The original contributions presented in the study are included in the article/Supplementary Material, further inquiries can be directed to the corresponding author.
